# Assessment of Ganglion Cell Complex and Peripapillary Retinal Nerve Fiber Layer Changes following Cataract Surgery in Patients with Pseudoexfoliation Glaucoma

**DOI:** 10.1155/2019/8162825

**Published:** 2019-09-08

**Authors:** Ahmed A. Abdelghany, Moataz A. Sallam, Abdallah A. Ellabban

**Affiliations:** Ophthalmology Department, Suez Canal University Hospitals, Ismailia 41111, Egypt

## Abstract

**Purpose:**

To assess eye pressure, ganglion cell complex, and retinal nerve fiber layer changes following cataract surgery in patients with pseudoexfoliation glaucoma.

**Methods:**

Eighty-five patients with pseudoexfoliation glaucoma (PEXG) were included in the study. They were divided into two groups; the first group included patients with PEXG and cataract who underwent phacoemulsification (pseudophakic group; *n* = 40 eyes). The second group included patients with PEXG without cataract (control group; *n* = 45 eyes). Both groups were on antiglaucoma treatment. IOP changes after surgery and the ganglion cell complex (GCC) and peripapillary retinal nerve fiber layer (pRNFL) thicknesses were evaluated in patients underwent cataract extraction surgery compared to controls that did not have cataract, nor underwent surgery. Both groups were followed up postoperatively for 18 months.

**Results:**

There was no difference in the mean age and glaucoma stage in both groups (*P*=0.242 and 0.70, respectively). In the pseudophakic group, the mean IOP significantly dropped from 20.43 ± 0.90 to 17.00 ± 2.75 mmHg at the end of the follow-up period (*P* ≤ 0.001). Slight decrease (≈3 *μ*m) was recorded in the mean GCC thickness of the pseudophakic patients from the baseline at the end of the follow-up period. This decrease was lower than that of the controls (≈5 *μ*m). No significant pRNFL changes were recorded all over the postoperative visits (88.78 ± 22.55 *μ*m at 3 months, 88.67 ± 23.14 *μ*m at 6 months, 87.62 ± 23.04 *μ*m at 12 months, and 87.32 ± 22.61 *μ*m at 18 months) as compared to preoperative value (90.28 ± 22.31 *μ*m) with *P*=0.335, 0.387, 0.158, and 0.110, respectively, or controls (89.69 ± 21.76 *μ*m, 88.73 ± 21.08 *μ*m, 87.33 ± 20.67 *μ*m, and 87.23 ± 20.54 *μ*m with *P*=0.850, 0.990, 0.951, and 0.984).

**Conclusion:**

Phacoemulsification and IOL implantation may aid in managing pseudoexfoliation glaucoma by lowering IOP and slowing the rate of GCC and pRNFL losses over a short-term period.

## 1. Introduction

Glaucoma is a leading cause of irreversible blindness, and about 80 million people worldwide are expected to be affected by glaucoma by the year 2020 [1]. Pseudoexfoliation (PEX) syndrome is an age-related microfibrillopathy systemic disease, caused by progressive accumulation and gradual deposition of extracellular material over various body tissues [2]. Findings of pseudoexfoliation syndrome include flaky exfoliative material on the anterior lens capsule and the angle of the anterior chamber, peripheral pupillary transillumination defects, and often pigment on the back of the cornea. Eyes with pseudoexfoliation possess a higher risk for developing glaucoma. Pseudoexfoliation glaucoma (PEXG) is considered the most common form of secondary open-angle glaucoma and develops secondary to PEX syndrome [3–5]. Compared to POAG, PEXG is often not well controlled with medical treatment, associated with progressive retinal nerve fiber layer loss, and often requires surgical treatment for better prognosis [6–8].

In the last few years, there has been a growing interest in retinal ganglion cell complex. It consists of three layers; the inner plexiform layer (IPL) made up of the ganglion cell dendrites, the ganglion cell layer (GCL) made up of the ganglion cell bodies, and the retinal nerve fiber layer (RNFL) made up of the ganglion cell axons. All three layers, collectively known as the ganglion cell complex (GCC), become thinner as the ganglion cells die from glaucoma, making it an ideal site for imaging and detecting early glaucomatous progression [9–12]. Bhagat et al. found that GCC is superior to peripapillary retinal nerve fiber layer (pRNFL) for detection of early glaucoma [13].

Current literature showed that phacoemulsification and posterior chamber intraocular lens (PC-IOL) reduced intraocular pressure in patients with coexisting cataract and glaucoma either POAG, PEX syndrome, or PEXG [14–16]. Previous studies indicated that the higher the preoperative IOP values, the greater the postoperative IOP reduction [16].

This study aims at assessing eye pressure and GCC changes following cataract extraction surgery in patients with pseudoexfoliation glaucoma.

## 2. Methods

This prospective study was conducted at the Department of Ophthalmology, Suez Canal University, in the period between June 2015 and December 2017. The current study included patients with PEXG who are under regular review in the eye clinic. The institutional review board and Ethics committee of Suez Canal University approved this study, which adhered to the tenets of the Declaration of Helsinki. An informed consent was obtained from each patient for enrollment in the study. The study participants were divided into 2 groups. The first group included patients with pseudoexfoliation glaucoma (PEXG) and cataract who had cataract surgery (pseudophakic group; *n* = 40 eyes of 30 patients). The second (control) group involved patients with PEXG who did not have cataract (control group; *n* = 45 eyes of 34 patients).

All subjects of both groups were on IOP-lowering drops treatment (dual or triple therapy). Duration of glaucoma was recorded for each patient. The inclusion criteria were age older than 50 years, open angle on gonioscopy, a refractive error of < ±4 diopters, and cataract grade 2 or 3 on Lens Opacification Classification System III [17]. PEXG was diagnosed based on the coexistence of the exfoliative material on the pupil margin or on the lens capsule in slit-lamp biomicroscopy. Only patients with early glaucoma, defined by Hodapp–Parrish-Anderson criteria for staging glaucoma [18] using Humphrey visual field testing, were enrolled in the study.

Exclusion criteria were dense cataract, any other type of secondary glaucoma such as angle-closure glaucoma, primary open-angle glaucoma, pigmentary glaucoma, inflammatory glaucoma, neovascular glaucoma, red cell glaucoma, or angle recession glaucoma. Eyes with coexisting macular disorder that may affect the visual field testing or optical coherence tomography (OCT) examination (such as diabetic retinopathy and age-related macular degeneration), or any previous intraocular surgery were excluded from the study. Patients with moderate or severe glaucomatous damage were excluded from the study. Patients who were previously treated with selective laser trabeculoplasty or other glaucoma surgeries were also excluded from the study.

At baseline, all patients had complete ophthalmic examination, including a review of medical history, slit-lamp biomicroscopy of anterior and posterior segments, gonioscopy, Goldmann applanation tonometry, ultrasound pachymetry, visual field testing, and spectral domain optical coherence tomography (SD-OCT). Detailed slit-lamp biomicroscopy was performed to assess pseudoexfoliation deposition on anterior capsule of the lens, type and grade of cataract, and the presence of phacodonesis or zonular dehiscence. A detailed optic nerve examination was performed to assess the level of glaucomatous changes. A calibrated Goldmann applanation tonometer (Shin-Nippon Inc., Tokyo, Japan) attached to the slit lamp was used to measure sitting IOP. The mean of 3 consecutive IOP readings was calculated. Visual field testing was performed using the 24-2 Swedish Interactive Threshold Algorithm (SITA) of the Humphrey Field Analyzer (Humphrey-Zeiss Systems, Dublin, CA). Visual field testing was considered reliable when the reordered fixation loss and false-positive and false-negative errors did not exceed 10%.

SD-OCT evaluation was performed following pupillary dilatation using tropicamide 0.5% eye drops by the same experienced technician. Pupillary dilatation was done to get better scans because patients were cataractous. For each eye, GCC scan centered on the macula was acquired using Optovue Avanti Widefield SD-OCT (Optovue Inc., version 0.9.3, CA, USA). The GCC scan covers an area of 7 × 7 mm centered on the fovea with a scan density of 512 (vertical) and 128 (horizontal) scans. GCC thickness was automatically measured by the software from the internal limiting membrane to the outer boundary of the inner plexiform layer. Circular papillary scan for peripapillary retinal nerve fiber layer (pRNFL) was used to measure the average nerve fiber layer thickness; superior, inferior, temporal, and nasal quadrant thicknesses and RNFL thickness in each clock hour vector was included in the analysis of the software of optic nerve head (ONH) analysis mode. Scans with motion artifact, poor quality, or incorrect segmentation were rejected, and repeat scans were acquired. The average GCC and pRNFL thicknesses were recorded at each follow-up visit.

Only patients with PEXG and cataract (pseudophakic group) had cataract surgery performed. Using sub-Tenon's or topical anesthesia, phacoemulsification was performed (Whitestar Signature, Abbott Medical optics Inc., USA) via clear corneal temporal 2.2 mm incision and in-the-bag intraocular lens implantation (AcrySof, SA60AT, Alcon Inc., USA). In all eyes, the viscoelastic was meticulously and completely removed at the end of the surgery to avoid postoperative IOP spikes [19]. Postoperative drug regimen included ofloxacin 0.3% eye drops 4 times daily for 1 week and prednisolone acetate 1.0% eye drops 4 times daily, tapered over 2 weeks. Following cataract surgery, all patients were reviewed at 3, 6, 12, and 18 months with repeat IOP, VF, and SD-OCT measurements. Patients in the control group were regularly reviewed at outpatient clinic at similar intervals (±2 weeks) with repeat measurements of IOP, VF, pRNFL, and GCC thicknesses.

Statistical analysis was performed with SPSS software (SPSS for Windows, version 20.0; SPSS Inc., Chicago, IL). Percentage, mean, and standard deviation were calculated for quantitative data. Independent *t*-test was used to compare means between the two groups. Multivariate analysis was referred to as post hoc test for multiple comparisons for observed means. The chi-square test was applied for the comparison of the categorical variables in both groups. A *P* value less than 0.05 was considered statistically significant.

## 3. Results

The mean age of the pseudophakic and control groups was 57.62 ± 5.00 and 56.36 ± 4.91 years, respectively. There was no significant difference between the two groups in terms of age, gender, central corneal thickness, MD, axial length, and duration of PEXG (*P* = 0.242, 0.625, 0.820, 0.070, 0.956, and 0.815, respectively).  Table 1 displays the subjects' preoperative clinical characteristics.

Changes in IOP after cataract surgery are recorded in Table 2. The mean preoperative (baseline) IOP of pseudophakic eyes was 20.43 ± 0.90 mmHg. At 18 months postoperatively, it significantly decreased to 17.00 ± 2.75 mmHg (*P* ≤ 0.001). Regarding the control group, no significant changes occurred in terms of the mean IOP along the first year follow-up when compared to baseline (*P*=0.185 at 3 months, 0.058 at 6 months, and 0.013 at 12 months). However, a significant increase in IOP in controls at 18 months was recorded (*P*=0.004). The baseline mean IOP showed significant differences (*P* ≤ 0.001) between the two groups (20.42 ± 0.90 mmHg in the pseudophakic group and 16.62 ± 1.00 in the control group) while the differences were not significant (*P*=0.469) at the end of follow-up visits (17.00 ± 2.75 mmHg and 17.35 ± 2.02 mmHg, respectively).

As presented in Table 3, the mean GCC thickness of patients in the pseudophakic group showed no significant loss till 6 months postoperatively (*P*=0.261 at 3 months and 0.168 at 6 months), and then it started to record a statistically significant decrease more at 12 and 18 months as compared to preoperative thickness (*P* ≤ 0.001 at 12 months and 18 months). However, the controls showed significant loss in the mean GCC thickness at all follow-up visits as compared to the baseline (*P* ≤ 0.001 at 3 months, 6 months, 12 months, and 18 months). However, there was no significant difference between both groups at follow-up visits (*P*=0.159 at 3 months, 0.165 at 6 months, 0.137 at 12 months, and 0.150 at 18 months), and the amount of GCC loss was more for the controls than for the pseudophakic patients.

In Table 4, the mean pRNFL thickness of patients in the pseudophakic group showed no significant loss all over the postoperative visits at 3, 6, 12, and 18 months as compared to baseline (*P*=0.335, 0.387, 0.158, and 0.110, respectively) However, the controls showed significant loss in the mean GCC thickness at 6 and 12 months of follow-up visits as compared to the baseline (*P*=0.007 and 0.006, respectively). By comparing both groups, there was no significant difference in pRNFL recorded at the follow-up visits (*P*=0.850 at 3 months, 0.990 at 6 months, 0.951 at 12 months, and 0.984 at 18 months). It is noted that the amount of pRNFL loss was more for the controls than for the pseudophakic patients.

## 4. Discussion

The current study evaluated the glaucomatous changes following cataract extraction surgery in patients with pseudoexfoliation glaucoma. Our observations confirmed that phacoemulsification leads to reduction in IOP. The mean postoperative IOP (17.00 mmHg) in the pseudophakic group at 18 months was significantly lower than the respective preoperative value (20.43 mmHg). It was also significantly lower than the mean IOP of the control group. The differences highlight the role of cataract surgery in IOP reduction.

The data obtained are consistent with other studies that reported better IOP reduction after phacoemulsification. Among studies including PEXG and POAG patients, Jimenez-Roman et al. retrospectively studied 88 medically controlled patients with PEXG (*n* = 44) and POAG (*n* = 44) who underwent phacoemulsification cataract surgery. Their results presented a 20.3% decrease in IOP, respectively, in patients with PEXG at 12 months of follow-up. For patients with POAG, this represents a 20.0% decrease in IOP [16].

Phacoemulsification possesses important implications for glaucoma control including a low surgical risk procedure, rapid recovery, decrease in IOP, and making the angle of the anterior chamber deeper. In addition to this, phacoemulsification maintains the anatomical integrity of the eye by saving the conjunctiva and sclera. This in turn decreases the risks of filtration failure if subsequent glaucoma surgery is indicated [20]. It has been suggested that phacoemulsification removes a source of pseudoexfoliative material (the anterior lens capsule) and clears pseudoexfoliative material and pigment debris from the anterior segment, specifically from the trabecular meshwork [21]. Patients with pseudoexfoliation possess greater IOP drop following phacoemulsification [20]. Additionally, it has been described that the volume of irrigation fluid used intraoperatively affects IOP response after phacoemulsification surgery in patients with pseudoexfoliation, thus emphasizing the idea that the procedure may clear exfoliation material from the outflow system [22, 23].

Our results reported a nonsignificant (*P* > 0.05) change in the mean GCC thickness during the postoperative 6 months of follow-up period. But there was more loss of GCC thickness at 12 and 18 months than that during the first 3 and 6 postoperative months. Compared to the control group, the losses are nonsignificant (*P* > 0.05) throughout the follow-up period.

The mean GCC thickness in the pseudophakic group dropped from preoperative value of 83.17 ± 13.71 *μ*m till 79.90 ± 11.33 *μ*m at the end of the follow-up period. This GCC loss is slower in the pseudophakic group than occurred in the control group as the mean GCC thickness dropped from baseline measurement of 88.19 ± 13.14 *μ*m to 83.44 ± 11.11 *μ*m at the end of follow-up visits. The mean percent rate of RGC loss was −4.4% per year as reported by Medeiros et al. [24]. This is higher than that recorded in the pseudophakic group, indicating that cataract extraction by phacoemulsification may add in slowing the rate of GCC loss for glaucoma patients. However, the GCC loss in our study group is higher than the age-related loss which is 0.2% [25].

Roh et al. [26] studied changes in the macular ganglion cell-inner plexiform layer (mGC-IPL) thickness after cataract surgery in glaucoma patients as compared to normal subjects. They found no significant difference in postoperative change in the mean mGC-IPL thickness between glaucomatous and normal eyes [26]. However, Sari et al. reported that mGC-IPL thickness significantly increased at 1 week and 1 month after cataract surgery. The authors suggested that surgically induced inflammation might affect ganglion cells, resulting in a postoperative increase in mGC-IPL thickness. They showed that the postoperative mGC-IPL thickness tended to return to preoperative values 3 months after surgery [27].

In our study, the GCC was thinner in patients in the pseudophakic group than in patients in the control group, suggesting that presence of cataract may add a factor in difficultly controlling PEXG.

Regarding pRNFL thickness, our study found that no significant loss (*P* > 0.05) occurred in the pseudophakic group after cataract removal at all follow-up visits as compared to preoperative value or compared to controls, while there was a significant loss of pRNFL at 12 and 18 months of follow-up in patients in the control group. These results suggested that pRNFL loss in patients underwent cataract extraction was less than controls. This in turn suggested that cataract extraction aided in slowing the rate of loss of pRNFL. Jha et al. reported that RNFL thinning is a significant predictor in assessing progression of glaucoma [28]. Pseudoexfoliation glaucoma causes thinning of RNFL [29]. Cataract extraction by better control of IOP could also control the rate of pRNFL loss.

It was noticed in our study that GCC change occurred earlier than pRNFL in both groups and this supported the fact that GCC might be as useful as peripapillary retinal nerve fiber layer (pRNFL) for detection of early glaucoma [12].

One of the limitations of our study is its small sample size and the fact that our study might need longer follow-up period. Also, we have studied only the total GCC thickness. Study of superior and inferior GCC thicknesses may improve our results. Poor scans were discarded, but the presence of cataract may affect the quality of OCT scans [30].

In conclusion, our study showed that cataract surgery can help in better IOP control in PEX eyes and may slow the rate of GCC and pRNFL losses. Therefore, early cataract surgery may be considered for the treatment of patients with a coexisting cataract and PEX glaucoma. However, further studies are necessary to determine the long-term effect of cataract surgery in eyes with PEX glaucoma.

## Figures and Tables

**Table 1:** Baseline characteristics of the study participants.

	Pseudophakic group	Control group	*P* value
*N*	40	45	
Age, years	57.62 ± 5.00	56.36 ± 4.91	0.242^a^
Male/female	19/21	19/26	0.625^b^
Axial length (mm)	23.84 ± 1.12	23.83 ± 0.98	0.956^a^
Central corneal thickness (*μ*m)	534.95 ± 12.04	534.36 ± 11.92	0.820^a^
MD (dB)	−3.77 ± 2.51	−4.84 ± 2.85	0.070^a^
Mean duration of glaucoma, years	3.53 ± 1.48	3.60 ± 1.45	0.815^a^

^a^Independent *t*-test and ^b^chi-square test.

**Table 2:** Pre- and postoperative intraocular pressure (IOP) changes in the pseudophakic and control eyes.

	IOP (mmHg) among the pseudophakic group	IOP (mmHg) among the control group	*P* value^b^
Baseline	20.43 ± 0.90	16.62 ± 1.00	≤0.001
At 3 months (*P* value^a^)	15.35 ± 1.03 (≤0.001)	16.69 ± 1.04 (0.185)	≤0.001
At 6 months (*P* value^a^)	15.40 ± 1.03 (≤0.001)	16.73 ± 1.05 (0.058)	≤0.001
At 12 months (*P* value^a^)	16.20 ± 2.41 (≤0.001)	17.04 ± 1.52 (0.013)	0.054
At 18 months (*P* value^a^)	17.00 ± 2.75 (≤0.001)	17.35 ± 2.02 (0.004)	0.469

^a^ *P* value, compared with values at baseline (paired *t*-test). ^b^*P* value, comparison of IOP changes in the pseudophakic group vs. control group, at baseline and follow-up visits (independent *t*-test).

**Table 3:** Pre- and postoperative average macular ganglion cell complex (GCC) thickness changes in the pseudophakic and control eyes.

	GCC (*μ*m) among the pseudophakic group	GCC (*μ*m) among the control group	*P* value^b^
Baseline	83.17 ± 13.71	88.19 ± 13.14	0.088
At 3 months (*P* value^a^)	82.95 ± 13.80 (0.261)	87.07 ± 12.98 (≤0.001)	0.159
At 6 months (*P* value^a^)	82.29 ± 13.41 (0.168)	86.30 ± 12.86 (≤0.001)	0.165
At 12 months (*P* value^a^)	80.86 ± 12.29 (≤0.001)	84.84 ± 12.09 (≤0.001)	0.137
At 18 months (*P* value^a^)	79.90 ± 11.33 (≤0.001)	83.44 ± 11.11 (≤0.001)	0.150

^a^ *P* value, compared with values at baseline (paired *t*-test). ^b^*P* value, comparison of average GCC changes in the pseudophakic group vs. control group, at baseline and follow-up visits (independent *t*-test).

**Table 4:** Pre- and postoperative average peripapillary retinal nerve fiber layer (pRNFL) thickness changes in the pseudophakic and control eyes.

	pRNFL (*μ*m) among the pseudophakic group	pRNFL (*μ*m) among the control group	*P* value^b^
Baseline	90.28 ± 22.31	91.20 ± 21.58	0.848
At 3 months (*P* value^a^)	88.78 ± 22.55 (0.335)	89.69 ± 21.76 (0.276)	0.850
At 6 months (*P* value^a^)	88.67 ± 23.14 (0.387)	88.73 ± 21.08 (0.082)	0.990
At 12 months (*P* value^a^)	87.62 ± 23.04 (0.158)	87.33 ± 20.67 (0.007)	0.951
At 18 months (*P* value^a^)	87.32 ± 22.61 (0.110)	87.23 ± 20.54 (0.006)	0.984

^a^ *P* value, compared with values at baseline (paired *t*-test). ^b^*P* value, comparison of average pRNFL changes in the pseudophakic group versus control group, at baseline and follow-up visits (independent *t*-test).

## Data Availability

The data supporting the results of the current article are available from the corresponding author upon request.

## References

[1] Quigley H. A., Broman A. T. (2006). The number of people with glaucoma worldwide in 2010 and 2020. *British Journal of Ophthalmology*.

[2] Ritch R., Schlötzer-Schrehardt U. (2001). Exfoliation syndrome. *Survey of Ophthalmology*.

[3] Realini T. (2016). *Pseudoexfoliation Glaucoma: Which Treatments Work Best?*.

[4] Schlötzer-Schrehardt U., Naumann G. O. H. (2006). Ocular and systemic pseudoexfoliation syndrome. *American Journal of Ophthalmology*.

[5] Alvarez L., García M., González-Iglesias H. (2015). LOXL1 gene variants and their association with pseudoexfoliation glaucoma (XFG) in Spanish patients. *BMC Medical Genetics*.

[6] Klemetti A. (1988). Intraocular pressure in exfoliation syndrome. *Acta Ophthalmologica*.

[7] Desai M. A., Lee R. K. (2008). The medical and surgical management of pseudoexfoliation glaucoma. *International Ophthalmology Clinics*.

[8] Konstas A. G., Allan D. (1989). Pseudoexfoliation glaucoma in Greece. *Eye*.

[9] Nouri-Mahdavi K., Nowroozizadeh S., Nassiri N (2013). Macular ganglion cell/inner plexiform layer measurements by spectral domain optical coherence tomography for detection of early glaucoma and comparison to retinal nerve fiber layer measurements. *American Journal of Ophthalmology*.

[10] Na J. H., Lee K., Lee J. R., Baek S., Yoo S. J., Kook M. S. (2013). Detection of macular ganglion cell loss in preperimetric glaucoma patients with localized retinal nerve fiber defects by spectral-domain optical coherence tomography. *Clinical & Experimental Ophthalmology*.

[11] Jeoung J. W., Choi Y. J., Park K. H., Kim D. M. (2013). Macular ganglion cell imaging study: glaucoma diagnostic accuracy of spectral-domain optical coherence tomography. *Investigative Opthalmology & Visual Science*.

[12] Michelessi M., Riva I., Martini E. (2019). Macular versus nerve fibre layer versus optic nerve head imaging for diagnosing glaucoma at different stages of the disease: Multicenter Italian Glaucoma Imaging Study. *Acta Ophthalmologica*.

[13] Bhagat P. R., Deshpande K. V., Natu B. (2014). Utility of ganglion cell complex analysis in early diagnosis and monitoring of glaucoma using a different spectral domain optical coherence tomography. *Journal of Current Glaucoma Practice with DVD*.

[14] Merkur A., Damji K. F., Mintsioulis G., Hodge W. G. (2001). Intraocular pressure decrease after phacoemulsification in patients with pseudoexfoliation syndrome. *Journal of Cataract & Refractive Surgery*.

[15] Hayashi K., Hayashi H., Nakao F., Hayashi F. (2001). Effect of cataract surgery on intraocular pressure control in glaucoma patients. *Journal of Cataract & Refractive Surgery*.

[16] Jimenez-Roman J., Lazcano-Gomez G., Martínez-Baez K. (2017). Effect of phacoemulsification on intraocular pressure in patients with primary open angle glaucoma and pseudoexfoliation glaucoma. *International Journal of Ophthalmology*.

[17] Chylack L. T., Wolfe J. K., Singer D. M. (1993). The lens opacities classification system III. The longitudinal study of cataract study group. *Archives of Ophthalmology (Chicago, III: 1960)*.

[18] Hodapp E., Parrish R. K., Anderson D. R. (1993). *Clinical Decisions in Glaucoma*.

[19] Fogagnolo P., Centofanti M., Figus M. (2012). Short-term changes in intraocular pressure after phacoemulsification in glaucoma patients. *Ophthalmologica*.

[20] Eid T. M. (2011). Primary lens extraction for glaucoma management: a review article. *Saudi Journal of Ophthalmology*.

[21] Kim Y. J., Kang M. H., Cho H. Y., Lim H. W., Seong M. (2014). Comparative study of macular ganglion cell complex thickness measured by spectral-domain optical coherence tomography in healthy eyes, eyes with preperimetric glaucoma, and eyes with early glaucoma. *Japanese Journal of Ophthalmology*.

[22] Chen P. P., Lin S. C., Junk A. K., Radhakrishnan S., Singh K., Chen T. C. (2015). The effect of phacoemulsification on intraocular pressure in glaucoma patients: a report by the American Academy of Ophthalmology. *Ophthalmology*.

[23] Damji K. F., Konstas A. G. P., Liebmann J. M. (2006). Intraocular pressure following phacoemulsification in patients with and without exfoliation syndrome: a 2 year prospective study. *British Journal of Ophthalmology*.

[24] Medeiros F. A., Zangwill L. M., Anderson D. R. (2012). Estimating the rate of retinal ganglion cell loss in glaucoma. *American Journal of Ophthalmology*.

[25] Hammel N., Belghith A., Weinreb R. N., Medeiros F. A., Mendoza N., Zangwill L. M. (2017). Comparing the rates of retinal nerve fiber layer and ganglion cell-inner plexiform layer loss in healthy eyes and in glaucoma eyes. *American Journal of Ophthalmology*.

[26] Roh H. C., Park C. Y., Kim M. (2016). Changes of the macular ganglion cell-inner plexiform layer thickness after cataract surgery in glaucoma patients. *Journal of Ophthalmology*.

[27] Sari E. S., Ermis S. S., Yazici A., Koytak A., Sahin G., Kilic A. (2014). The effect of intracameral anesthesia on macular thickness and ganglion cell-inner plexiform layer thickness after uneventful phacoemulsification surgery: prospective and randomized controlled trial. *Graefe’s Archive for Clinical and Experimental Ophthalmology*.

[28] Jha B., Sharma R., Vanathi M. (2017). Effect of phacoemulsification on measurement of retinal nerve fiber layer and optic nerve head parameters using spectral-domain-optical coherence tomography. *Oman Journal of Ophthalmology*.

[29] Demircan S., Yılmaz U., Küçük E. (2017). The effect of pseudoexfoliation syndrome on the retinal nerve fiber layer and choroid thickness. *Seminars in Ophthalmology*.

[30] Mwanza J. C., Bhorade A. M., Sekhon N. (2011). Effect of cataract and its removal on signal strength and peripapillary retinal nerve fiber layer optical coherence tomography measurements. *Journal of Glaucoma*.

